# Implementation of an efficacious intervention for high risk women in Mexico: protocol for a multi-site randomized trial with a parallel study of organizational factors

**DOI:** 10.1186/1748-5908-7-105

**Published:** 2012-10-29

**Authors:** Thomas L Patterson, Shirley J Semple, Claudia V Chavarin, Doroteo V Mendoza, Lorena E Santos, Mark Chaffin, Lawrence A Palinkas, Steffanie A Strathdee, Gregory A Aarons

**Affiliations:** 1 Department of Psychiatry, University of California at San Diego, 9500 Gilman Drive La Jolla, California 92093, USA; 2 Research Department, Mexican Foundation for Family Planning, (Mexfam), Juarez 208, Tlalpan, Mexico, D.F, 14000, Mexico; 3 Department of Pediatrics, University of Oklahoma, PO Box 26901, Oklahoma City, OK, USA; 4 School of Social Work, University of Southern California, University Park, Los Angeles, California, 90089, USA; 5 Department of Medicine, University of California at San Diego, 9500 Gilman Drive, La Jolla, California, 92093, USA

**Keywords:** Implementation, Dissemination, HIV, Evidence-based intervention, Female sex workers

## Abstract

**Background:**

Studies of implementation of efficacious human immunodeficiency virus (HIV) prevention interventions are rare, especially in resource-poor settings, but important, because they have the potential to increase the impact of interventions by improving uptake and sustainability. Few studies have focused on provider and organizational factors that may influence uptake and fidelity to core intervention components. Using a hybrid design, we will study the implementation of an efficacious intervention to reduce sexually transmitted infections (STIs) among female sex workers (FSWs) in 12 cities across Mexico. Our protocol will test a ‘train-the-trainer’ implementation model for transporting the Mujer Segura (Healthy Woman) intervention into community-based organizations (CBOs).

**Methods:**

We have partnered with Mexican Foundation for Family Planning (Mexfam), a non-governmental organization that has CBOs throughout Mexico. At each CBO, trained ethnographers will survey CBO staff on characteristics of their organization and on their attitudes toward their CBO and toward the implementation of evidence-based interventions (EBIs). Then, after CBO staff recruit a sample of 80 eligible FSWs and deliver a standard-care, didactic intervention to 40 women randomly selected from that pool, a Mexfam staff person will be trained in the Mujer Segura intervention and will then train other counselors to deliver Mujer Segura to the 40 remaining participating FSWs. FSW participants will receive a baseline behavioral assessment and be tested for HIV and STIs (syphilis, gonorrhea, and chlamydia); they will be reassessed at six months post-intervention to measure for possible intervention effects. At the same time, both qualitative and quantitative data will be collected on the implementation process, including measures of counselors’ fidelity to the intervention model. After data collection at each CBO is complete, the relative efficacy of the Mujer Segura intervention will be analyzed, and across CBOs, correlations will be examined between individual and organizational provider characteristics and intervention efficacy.

**Discussion:**

This cooperative, bi-national research study will provide critical insights into barriers and facilitating factors associated with implementing interventions in CBOs using the ‘train the trainer’ model. Our work builds on similar scale-up strategies that have been effective in the United States. This study has the potential to increase our knowledge of the generalizability of such strategies across health issues, national contexts, and organizational contexts.

**Trial registration:**

NCT01465607

## Background

The Joint United Nations Programme on human immunodeficiency virus/ acquired immunodeficiency syndrome (HIV/AIDS) estimates that in 2009, 2.6 million new infections and 1.8 million AIDS deaths occurred and almost 33.3 million adults and children were living with HIV or AIDS worldwide, with the great majority of them in low- and middle-income countries [1]. Global HIV prevention efforts have focused on various risk populations, including female sex workers (FSWs). Our research suggests that HIV prevalence is rising rapidly among FSWs on the Mexico–United States (US) border. In 1991, HIV prevalence in a sample of 415 FSWs in Tijuana was 0.5% [2], but in a more recent study of 924 FSWs in Tijuana and Ciudad Juarez, HIV prevalence was 6% [3], and HIV incidence in the control group was 2 per 100 person-years (py) [4].

Our research team developed a brief (35-minute) behavioral intervention to promote condom use and enhance safer sex negotiation skills among FSWs in Mexican border cities. This project, known as Mujer Segura (Healthy Woman), was recently shown to be efficacious [4], mediated by improvements in FSW self-efficacy [5]. FSWs randomized into Mujer Segura had a 40% decline in cumulative incidence of sexually transmitted infections (STIs). Incidence density for the intervention versus control decreased significantly: 13.8 versus 24.92 per 100 py for STIs combined (p = 0.03) and 0 versus 2.01 per 100 py for HIV (p<0.001), with concomitant increases in total numbers and percentages of protected sex acts and decreases in total numbers of unprotected sex acts with clients (p<0.05) at six-month follow-up [4].

Federal health authorities in Mexico encouraged us to formally evaluate the implementation process for Mujer Segura to facilitate its implementation throughout the country. Implementation studies in the HIV literature have failed to focus on provider and organizational factors that may influence uptake and fidelity to core intervention components. We assembled a bi-national team of experts in implementation studies and HIV prevention and are preparing to test an implementation model for transporting Mujer Segura into existing community-based organizations (CBOs) in Mexico.

Our implementation model aims to develop local and culturally relevant expertise and infrastructure to sustain and further disseminate Mujer Segura upon completion of this project. Partnering with the Mexican Foundation for Family Planning (Mexfam), a non-governmental organization (NGO) that has sites throughout Mexico, we will examine whether our implementation model can develop a network of HIV/STI prevention services for FSWs with self-sustaining levels of model fidelity and provider competency. Our findings could generalize not only to future implementation efforts involving Mujer Segura but also to the implementation of other evidence-based interventions in resource-constrained settings.

The specific aims of this study are to**:** determine if our implementation model can achieve high levels of intervention fidelity and provider competency in the context of a large-scale implementation effort; characterize the relationship between individual provider characteristics and organizational factors and determine their impact on the implementation of Mujer Segura using a mixed-methods (quantitative and qualitative) approach; determine whether the implementation of Mujer Segura by CBOs is associated with decreased sexual risk behaviors among Mexican FSWs over a six-month period (*e.g.*, increased condom use with clients, reductions in STI incidence); and determine whether improvements in sexual risk behaviors among Mujer Segura FSWs are associated with variations in intervention fidelity and counselor competency.

## Methods and design

This study is a Hybrid Type 2 design that simultaneously tests a clinical intervention and an implementation strategy [6]. This study has two levels. The first level involves a multi-site, randomized controlled trial (RCT), with a two-arm, parallel design and a 50/50 allocation ratio, of a safer-sex intervention for female sex workers (FSWs) that has been shown efficacious in a previous RCT [4]. Twelve different CBOs in different parts of Mexico will each enroll and randomize 80 FSWs in this RCT. The second level of the study, which is not randomized, involves the collection and analysis of data at the intervention sites concerning organizational factors that are hypothesized to affect the efficacy of the intervention. Conduct of the study began in January, 2011 in Mexico City, and we expect to complete recruitment and data collection at all sites by the end of 2015.

### Level one study: RCT of mujer Segura intervention

Eligibility criteria for participation in the RCT of the safer-sex intervention are the same as those of the previous efficacy study. Participants must be: biologically female; at least 18 years of age; self-identify as a female sex worker; report having traded sex for drugs, money, shelter, or other material benefit within the previous two months; have had unprotected vaginal or anal sex with a client at least once during the previous two months; have no previous HIV-positive test result; and agree to be tested for HIV and STIs at baseline and at six-month follow-up.

As noted, each study site will screen interested local FSWs for eligibility until 80 eligible women have been found and agree to participate. The number of FSWs to be recruited at each site (n = 80) was determined using effect sizes from the parent project (see below under Analyses). Our data center computer-generated a random order for intervention group assignment (40 participants to the Mujer Segura intervention and 40 to a standard care counseling condition). To minimize the potential influence of knowledge of Mujer Segura upon the treatment of the comparison condition, staff will assess and counsel the 40 comparison condition FSWs before being trained in Mujer Segura*;* the participants assigned to Mujer Segura will be put on a waitlist and contacted later to schedule their baseline assessment and counseling session. For both groups, the baseline visit will consist of a psychosocial and behavioral questionnaire lasting approximately 35 minutes, collection of blood and urine samples for laboratory testing (HIV, syphilis, gonorrhea, chlamydia), and the counseling intervention. The questionnaire will be administered using computer-assisted personal interviewing (CAPI). Women found to be HIV-positive at baseline will be counseled regarding their HIV test result, referred for treatment, and withdrawn from the study. The CAPI assessment and the HIV and STI tests will be repeated at six months following the counseling intervention. Women presenting with active syphilis, gonorrhea, or chlamydia at baseline will be treated for those conditions to permit distinction of incident from prevalent cases at six-month follow-up.

To the greatest extent possible, participants will be blinded to their allocation. Although the waitlist control design that we have adopted will reveal the existence of two different interventions, study staff will not reveal to participants which one is experimental and which is the control, and our experience suggests that little communication between FSWs is likely to occur. Likewise, the CAPI assessments will be administered by outreach workers rather than counselors, to minimize the assessors’ knowledge of intervention assignment. Because the delayed group might experience higher attrition, we will conduct follow-up activities that have proved effective in our earlier studies [4]. We will also re-screen the delayed participants to ensure that their recent risk behavior (*i.e.*, within the past two months) has not diminished to the point of disqualifying them. Finally, we will compare data between the two groups to determine if dropouts differ by demographic variables, risk behaviors, or psychosocial factors.

### Description of intervention conditions

#### Experimental

Mujer Segura is a brief (35 to 40 minute), single-session, individual intervention that combines principles of motivational interviewing (MI), social cognitive theory (SCT), and the theory of reasoned action [7,8]. A detailed description is provided elsewhere [4]. The counselor uses motivational interviewing techniques (*e.g.*, key questions, reflective listening, summarization, affirmation, and appropriate use of cultural cues) to increase the participant’s motivations to practice safer sex.

#### Comparison

Because FSWs are at high risk of STI and HIV infection, both interventions impart knowledge necessary for practicing safer sex. However, in the standard counseling condition, topics are covered in a lecture format without the use of MI or the role plays, exercises, and problem-solving emphasized in the Mujer Segura intervention. The comparison condition is time-equivalent and utilizes counseling materials provided CENSIDA, the Mexican federal agency responsible for HIV and AIDS prevention [9], that are currently in use at the CBOs.

### Measures for level 1 study

The primary outcomes for the RCT of the Mujer Segura safer-sex intervention with FSWs are decreases in sexual risk behavior (greater frequency of condom use, lower number and proportion of unprotected sex acts, decreased incidence of HIV and STIs). Secondary outcomes are increases in knowledge, self-efficacy, and outcome expectancies regarding safer sex.

### Behavioral and psychosocial questionnaire (CAPI)

This assessment, which was used with over 1,000 FSWs in our previous study of Mujer Segura*,* covers the following domains:

1. Background characteristics such as socio-demographics, family variables (*e.g.*, number of children), and financial need (*e.g.*, number of dependents).

2. Contextual factors such as work setting (*e.g.*, street, brothel), relationship with pimp or manager (*e.g.*, control over client selection), client characteristics, demands for unprotected sex, amount received for protected versus unprotected sex, and availability of condoms and sterile syringes.

3. Substance use includes current practices and history (*e.g.*, age at first use of alcohol and specific drugs), amount of alcohol consumed [10], types of substances used alone and in combination, routes of administration, injection practices, and environmental influences.

4. Sexual risk behaviors will include number and frequency of unprotected sex acts (vaginal, oral, and anal) with clients and with spouse or steady partner(s); number of clients (regular and nonregular); number and type of other sex partners (non-clients); number of partners who inject drugs.

5. Mechanisms of change: Consistent with our theoretical framework, we include measures of knowledge, attitudes, intentions, and peer norms about safer sex. Knowledge will be measured with an 18-item scale that assesses awareness of the importance of condom use with respect to HIV/STI prevention [11]. Attitudes are assessed with the Self-Efficacy Towards Condom Use scale, a five-item measure that asks FSWs the extent to which they are able to use a condom properly with clients. Peer norms about HIV prevention through safer sex will also be measured [12]. Outcome expectancies will be measured by six items.

### Testing for HIV and STIs

The Advanced QualityTM Rapid Anti-HIV (1 and 2) test is a colloidal gold-enhanced, immunochromatographic assay for the qualitative detection of HIV antibodies in whole blood, serum, or plasma, which we will use at baseline and six-month follow-up to ascertain HIV serostatus of FSW participants. All reactive samples will then be tested using HIV-1, 2 serum antibody enzyme immunoassay (EIA) and indirect fluorescent antibody (IFA) tests. While the incidence of seroconversion is likely to be low over a six-month period, these are important descriptive data that may vary across cities. FSWs will also be screened for syphilis, chlamydia, and gonorrhea. Syphilis serology will include a rapid diagnostic screening for the qualitative detection of antibodies to *Treponema pallidum* in serum, plasma or whole blood. All reactive samples will be subjected to the rapid plasma reagin (RPR) test and the *T. pallidum* particle agglutination assay (TPPA). Urine samples will be collected using the Gen-Probe Aptima Combo 2® Assay for *C. trachomatis* and *N. gonorrhoeae*. HIV/STI test results will be provided to participants by nurses within two weeks of testing. Those testing HIV-positive will be referred to their municipal clinic for free medical care, and those who test positive for another STI will be treated on-site at the Mexfam CBO. HIV/STI reporting is mandatory throughout Mexico, and requirements are consistent across states. Reporting requirements are detailed in the FSW consent form along with possible adverse consequences (*e.g.*, loss of license to practice sex work if testing positive for HIV).

### Level two study: mixed-methods analysis of organizational and provider characteristics

#### Site selection

The selection of the study sites was guided by considerations of geographic distribution and organizational capacity. Preliminary analyses suggested that 12 implementation sites (CBOs) would be needed to achieve adequate statistical power to analyze the relationships of personal provider and organizational characteristics to the efficacy of the intervention. To ensure a representative characterization of each site, as well as the relevance of the data to the efficacy of the implementation, we will secure the cooperation of approximately 12 staff members per site to participate in qualitative interviews and quantitative assessments of their attitudes and beliefs toward their work and their organization as well as toward evidence-based interventions. These personnel will include one internal trainer, approximately six to eight persons qualified to deliver the intervention, two supervisors, and the local CBO administrator.

The CBOs chosen for this implementation study are all part of the Mexfam*.* Headquartered in Mexico City, Mexfam is a non-profit, non-governmental organization that operates sexual and reproductive health programs in 22 states in Mexico. Among its other community health programs, Mexfam has worked to increase HIV prevention through advocacy, gender- and culture-sensitive interventions, and educational media campaigns [13]. To make the sample of sites representative, Mexfam included CBOs with varying capacities, sizes, and geographic locations. Each site had to meet the following minimum criteria: a staff member qualified to be an internal trainer; a core of approximately six to eight high-potential, stable staff members who could be trained as intervention counselors; an organizational culture that supports innovation and evidence-based perspectives; a strong positive reputation in its community; the capacity to deliver professional peer-to-peer training; strong cultural competency and willingness to work with FSWs. From 23 eligible clinics, 12 were randomly drawn for participation in this study.

Prior to commencing the project, our research team developed an instrument to assess the capacity of the 12 selected CBOs to implement the Mujer Segura intervention*.* Indicators were derived from the CDC’s six-domain model [14]. Possible scores ranged from 0 (no capacity) to 150 (100% capacity). Among our 12 sites, preparedness scores ranged from 57 to 115 (Mean = 89.3, SD=15.18, Median = 94.5). As evidenced by these data, the participating CBOs are characterized by varying degrees of agency capacity.

#### Implementation process

**Figure 1:**
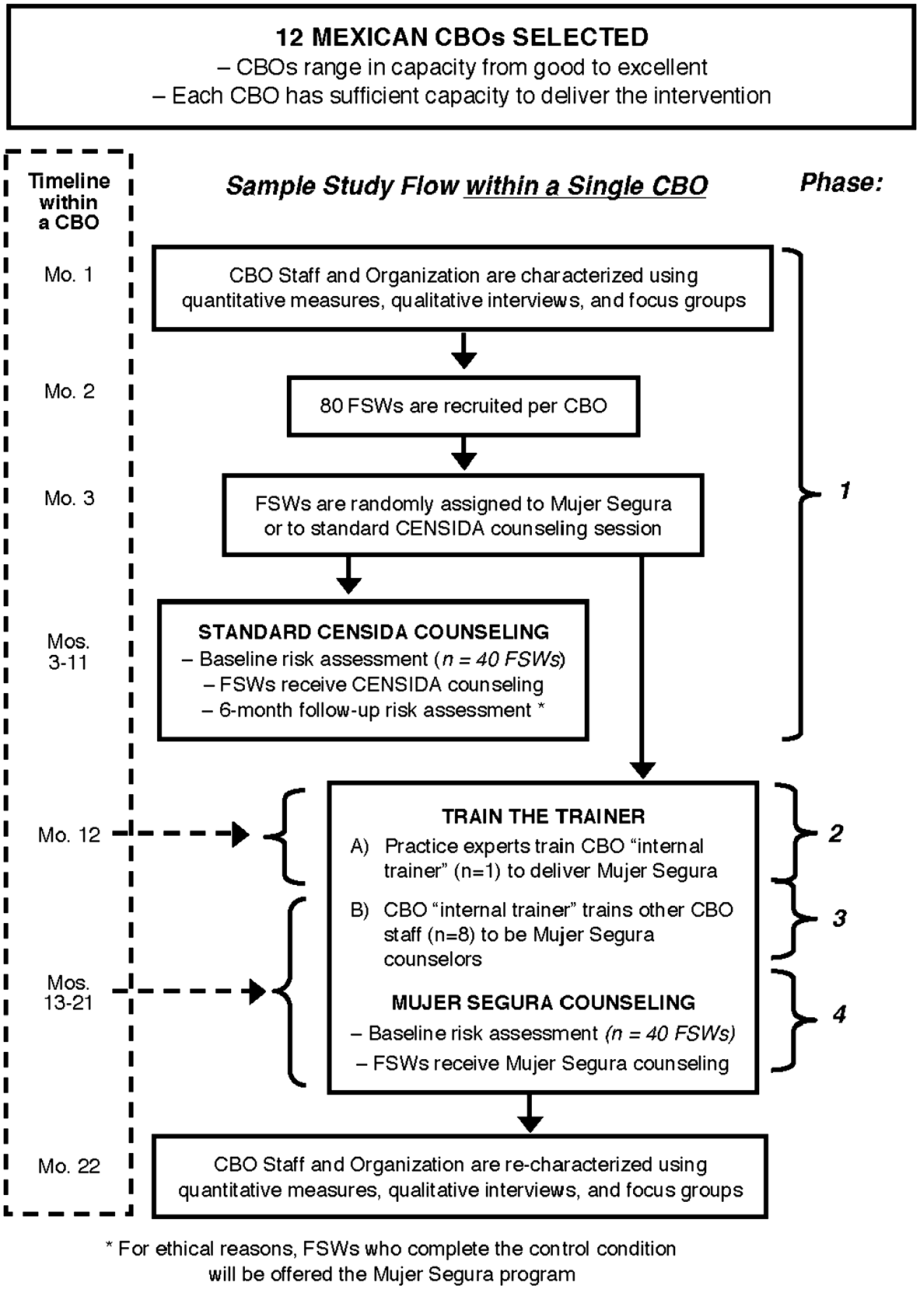
Study flow at participating implementation sites.

The model for this project is a ‘train-the-trainer’ approach that will proceed through four phases (see Figure 1). In phase one, selected CBO staff will respond to questionnaires measuring provider and organizational factors and participate in qualitative interviews and focus groups conducted by ethnographers. CBO outreach workers will subsequently recruit 80 FSWs as described above. FSWs assigned to the standard care condition will then be assessed and receive their intervention.

In phase two, a CBO staff member will be selected to become the organization's ‘internal trainer.’ This person will receive intensive training in the Mujer Segura intervention by the developers of the model and by ‘practice experts’ (counselors who delivered Mujer Segura in our earlier study). In this phase, based on feedback from CBO staff, the model developers and practice experts will make any necessary cultural adaptations. Internal trainers will practice delivering the intervention while being coached *in vivo* by the model developers and practice experts. Phase two ends when the internal trainer meets strict criteria of proficiency and is formally certified.

In phase three, additional CBO staff persons will receive Mujer Segura training from the internal trainer. When CBO counselor trainees meet criteria for practice competency, outreach workers will administer baseline behavioral assessments to the 40 FSWs randomly assigned to Mujer Segura*,* after which the trained CBO counselors will begin to deliver the intervention. Fidelity to the intervention protocol will be monitored in three ways: through direct observation by the internal trainer, and through the completion of fidelity checklists by both FSW participants and the counselors themselves.

In phase four, internal trainers will shift to a maintenance function, which will include training and coaching new hires and a less-intensive schedule of *in vivo* coaching and monitoring of counselors’ fidelity to the intervention model.

#### Training philosophies and procedures

A key feature of the proposed implementation model is its ‘train-the trainer’ approach. This approach is considered a good choice for agencies with limited financial resources, such as CBOs and non-profit organizations [15]. In the context of sexual risk reduction counseling, train-the-trainer involves identifying a staff member who has some expertise in HIV/STI counseling and teaching that person how to train other staff in delivery of the counseling program. An effective train-the-trainer program teaches leadership and coaching skills, such as how to facilitate a meeting and how to assist staff who are having difficulties in delivering the intervention [15]. It is important to the success of this model that the internal trainer’s duties not simply be added to his or her existing responsibilities [15]. The train-the-trainer approach can lead to a gradual degeneration of skills, but the availability of detailed counseling manuals and training materials will help to mitigate this concern.

#### Training the internal trainer

The implementation plan is built on four fundamental principles:

1. Developing local expertise in the delivery of Mujer Segura by certifying a skilled internal trainer is important for sustaining competency and fidelity to the intervention model.

2. Direct coaching and modeling in the actual practice setting are critical for optimal acquisition of skills.

3. It will be critical to monitor and maintain fidelity. This will be achieved through regular observation by the internal trainer. Adaptations to the local context or gradual improvements in the model should be carefully planned, transparent, and systematically implemented.

4. Policy leadership and funding structures are important for institutionalizing the new practice model.

#### Training CBO staff

After the comparison-condition FSWs have been assessed and counseled and the internal trainer has been certified competent by the practice experts, the internal trainer will provide CBO staff with intensive training in the Mujer Segura protocol. In order to ‘pass’ the training, counselors must achieve a minimum score of 90% on quizzes. After that, each trainee will be observed and coached by the internal trainer for a full week (five days) in the delivery of the intervention to FSWs. After demonstrating fidelity to the model in five sessions (without prompts), trainees will be allowed to counsel FSWs without supervision.

### Measures for the level two study

**Figure 2:**
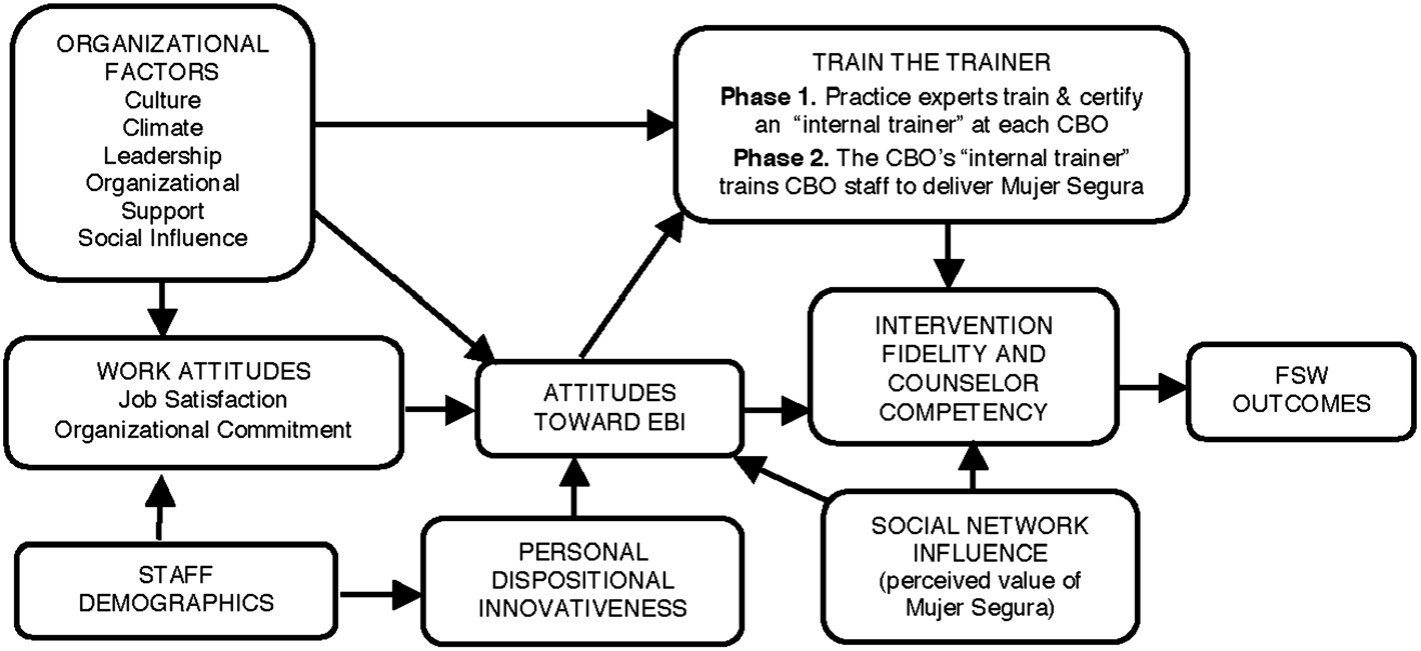
Organizational and provider factors influencing uptake, fidelity, and outcomes of evidence-based interventions (adapted from Aarons, 2005).

Organizational and provider factors can significantly affect the implementation of an evidence-based intervention (Figure 2). For example, social influences within an organization can affect employee attitudes toward the adoption of new practices [16]. Moreover, willingness to adopt a new model can be influenced by an organization’s culture and climate and by individual providers’ characteristics (such as job tenure) [17]. In this research, measures that will be administered to CBO personnel tap into three main theoretical constructs (two of which are similarly named): social influence, social network influence, and ‘personal dispositional innovativeness.’

Measures of social influence include the short-form Group Innovation Inventory (GII), which measures the influence of other providers in the CBO [16], and Siegel’s Scale of Support for Innovation (SSSI), which assesses staff members’ perceptions of their organization’s support for innovation and change [18]. Subscales of the Organizational Social Context Survey [19] will be used to assess aspects of organizational structure and functioning that have been shown to be associated with attitudes toward EBIs [17,20,21]. Finally, we will use the Multifactor Leadership Questionnaire (MLQ) [22] to assess characteristics of organizational leadership [23].

Individual providers can also be influenced in their attitudes about specific innovations by the perceptions of how well known and valued those innovations are by their colleagues and professional contacts. We call this phenomenon social network influence, and have adapted a ‘network externalities’ scale to assess the extent of knowledge about Mujer Segura and perceptions of its utility among service providers’ peers [24-26].

Personal dispositional innovativeness will be measured with the ‘adaptability’ and ‘conscientiousness’ subscales of the Emotional Competency Inventory (ECI) [27]. In addition, we will use the Evidence-Based Practice Attitude Scale (EBPAS), developed by Aarons, to measure individual providers’ attitudes toward the adoption of evidence-based practices (EBP) [21,28].

Finally, a questionnaire adapted by Dr. Aarons will be used to assess staff demographics and patient contact (*e.g.*, age, race, education, primary discipline, experience, caseload, frequency of contact with FSW clients). Demographics have been shown to influence attitudes toward adopting EBIs [21].

All assessments of staff will be administered at project initiation (pre-implementation) and after completion of implementation at each site. Data from CBO staff will be collected using CAPI technology. In their consent form, CBO staff will be informed that participation is voluntary and that they can withdraw at any time or refuse to answer any question without penalty. Staff will also be assured that their survey data will be kept confidential and will not be used for evaluation of job performance. Furthermore, only aggregate or grouped data on staff will be provided to CBO management. Staff participants will receive an incentive of $10 USD for the CAPI survey at both baseline and follow-up, and they will receive $10 for both the semi-structured interview and focus group at both time points, for a possible total of $60.

### Qualitative methods

We anticipate that qualitative data will help explain differences in FSW outcomes by CBO site. Our mixed-methods approach will gather data from *in vivo* observation of Mujer Segura counseling sessions, semi-structured interviews with CBO personnel, and separate focus groups with CBO counselors and supervisors both before and after implementation of Mujer Segura. Qualitative methods will be used to: identify factors that contribute to fidelity to the intervention protocol; identify organizational characteristics that facilitate or hinder adoption of the intervention model; compare and contrast the barriers and resources identified by agency directors, supervisors, and counselors; and develop a heuristic model integrating the perspectives of all stakeholders and generate hypotheses regarding processes and implementation outcomes.

### Ethnographic observation

At each CBO, an ethnographer will observe the training of the internal trainer, the training of CBO staff by the internal trainer, and Mujer Segura counseling sessions conducted by CBO staff. The ethnographer will prepare detailed notes consisting of observations, impressions, and notations on methods.

### Semi-structured interviews

The ethnographer will conduct a semi-structured interview, lasting approximately 60 minutes, with each participating CBO staff member. These interviews will address a subset of organizational characteristics defined as operationally salient in the implementation model. Questions will elicit information on: knowledge, attitudes, and behavior related to the intervention; characteristics of the agency and team; characteristics of the external environment; and (in the follow-up interview) characteristics of the implementation process. Questions are sufficiently open-ended to allow respondents to elaborate on issues they consider important or relevant. Sample questions include: How effective was the training in terms of building skills in the practices? What could be done better? How effective were the supervision and coaching in building skills in the practices? Interviews will be audio-recorded and transcribed for analysis. Data gathered through the semi-structured interviews will be used to develop a conceptual model that diagrams the major variables identified as influencing implementation of the intervention.

### Focus groups with CBO staff

At each CBO, we will conduct focus group sessions with counselors who delivered Mujer Segura and with CBO management (director, supervisors)*.* The discussions will last less than two hours, be audio-recorded, and follow a ‘funnel’ structure, starting with broader issues and narrowing down to more specific, participant-driven illustrations. Examples of issues that will be addressed in the focus group with counselors include: Are there any features of your organization that you feel were not included or given insufficient emphasis in the questionnaires you filled out? Do you feel any of these features are likely to affect your willingness or ability to change the way you deliver the intervention?

### Integration of qualitative and quantitative methods

Mixed methods such as those proposed for this project have been recommended for the study of implementations of innovations [29-31]. Our design incorporates three components for the integration of qualitative and quantitative data. The first is the corroboration of quantitative findings of organizational influences with data collected through semi-structured interviews and focus groups, a process known as triangulation [32]. Triangulation will be especially important in the proposed study, because sample size (especially at the level of the basic organizational units, the CBO) may limit statistical power to test some hypotheses. Findings from the qualitative studies can be used to assess validity (external as well as internal) of EBI measures under these circumstances.

The second component of our mixed-methods design is complementary to the administration of quantitative measures, which provide a starting point of inquiry in the semi-structured interviews, and conversely, enhance the validity of the measurement instruments by expanding upon domains of content.

The third component is ‘expansion,’ in which the qualitative conclusions about the implementation process that are derived from the semi-structured interviews and focus groups are presented side-by-side with quantitative results to determine whether the two methods of data collection lead to similar conclusions concerning barriers and facilitators of implementation [33]. Given the unit of analysis (the CBO), there are power limitations for some research questions. However, the integration of qualitative and quantitative methods helps to mitigate this concern. In addition, the use of widely different methodological approaches will serve either to increase confidence in hypotheses or to guide the research team to modify the model for later testing of alternative hypotheses.

### Assessing fidelity of training and of intervention delivery

Qualitative data that focus on fidelity, deviations from fidelity, and associated factors will be gathered by an ethnographer. Detailed field notes consisting of observations, impressions, and notations pertaining to Mujer Segura counseling sessions will be gathered. In addition, the ethnographer will conduct focus group interviews and semi-structured interviews with CBO staff (counselors, internal trainer, supervisors, director) at baseline (pre-implementation) and after completion of delivery of Mujer Segura to all 40 FSWs assigned to that condition. The ethnographer will also gather qualitative data (*e.g.*, field notes) and complete a fidelity checklist to document the training of the CBO’s internal trainer and the subsequent training and coaching of CBO staff by the internal trainer. Because the focus of this study is the process of implementing the efficacious Mujer Segura intervention, the formal counseling sessions will be audio-recorded and scored for fidelity throughout the standard counseling and Mujer Segura counseling period.

### Analysis

Aim one: Determine if our implementation model can achieve high levels of intervention fidelity and provider competency in the context of a large-scale implementation effort

A form of equivalence testing [34-37] known as non-inferiority [38,39] will be used to evaluate the aim one hypothesis. Given the nested data structure (*i.e.*, FSWs nested in intervention conditions nested in CBOs), mixed linear models will be used to adjust standard errors for possible dependency among observations. The outcome in aim one focuses on Mujer Segura fidelity. We expect that CBO counselors will attain approximately 90% treatment fidelity. Power calculations for non-inferiority tests proposed for aim one were conducted using SAS® Proc Power. To calculate power, we used a hypothesized difference of zero between population group means and a standard deviation of 15 in each group. The total sample size is 360 for CBO counselors (12 CBOs x 6 counselors per CBO x 5 FSWs per counselor). Given these parameters, power to test hypotheses of non-inferiority is >0.99.

Aim two: Characterize the relationship between individual provider characteristics and organizational factors and determine their impact on the implementation of Mujer Segura using a mixed-methods approach

In order to examine specific components of relationships in our model, analyses will utilize mixed linear models in order to adjust standard errors for possible dependency among observations. Significance tests will use an alpha of 0.05 (two tailed). Analyses will consist of regression and tests of mediation. Analyses will be informed by the conceptual model for public sector implementation proposed by Aarons and colleagues [40] and adapted for this study, in which organizational and provider characteristics affect provider attitudes, fidelity and outcomes during the implementation of an EBI [41,42]. Conversely, introduction of an EBI can modify the characteristics of an organization and—to a lesser extent—individual providers [41]. In particular, we will examine changes in processes within the organization and group process characteristics as a function of Mujer Segura implementation. We will also examine the relationship between specific organizational factors and provider characteristics, and their impact on the implementation of Mujer Segura. In order to examine social networks and social influence, we will examine the extent to which CBO counselors learn (from sources other than their formal training in the protocol) about Mujer Segura and also their perceptions of the intervention’s utility. We will then examine whether more knowledge and higher levels of perceived utility are associated with better fidelity and outcomes. In all CBOs, we will examine the degree to which leadership and organizational support are associated with more positive attitudes toward EBIs, fidelity, and outcomes. We will also examine the degree to which personal dispositional innovativeness and other provider characteristics such as age, education level, experience, and job tenure are associated with attitudes toward EBIs, fidelity, and FSW outcomes. We hypothesize that organizational characteristics and individual provider characteristics will have complex and reciprocal dynamics. We also propose that provider relationships and knowledge about Mujer Segura will be associated with attitudes toward EBIs. Further, we hypothesize that provider characteristics will be associated with fidelity to the intervention protocol.

Power was calculated under the assumptions of independent and dependent observations for the proposed correlation, regression, and mediation tests [43]. Under assumptions of independence, the sample size used was the total of 96-and-under dependence (ICC = 0.03), and a functional sample size of 79.34 was used. We estimated power of 0.80.

Aim three: Determine whether the implementation of Mujer Segura by CBOs is associated with decreased sexual risk behaviors among FSWs over a six-month period.

Aim four: Determine whether improvements in sexual risk behaviors among Mujer Segura FSWs are associated with variations in intervention fidelity and counselor competency

Due to potential non-independence of FSWs within each CBO, mixed linear models will be used to determine if participation in the Mujer Segura intervention was efficacious in improving sexual behavior outcomes (*e.g.*, condom use) compared to the treatment-as-usual condition. Within this model, intervention condition, time, and the intervention-by-time interaction will be entered as our primary independent variables. Because participants will be nested within CBOs, this factor will be entered as a random effect. A first-order autoregressive process (a stationary AR [1] process) will be used to characterize the autocorrelation structure.

Our second set of analyses for aim three will examine efficacy of the intervention for reducing incident HIV and other STIs. We will compare intervention groups in terms of cumulative incidence and incidence density. In both cases, the numerator will be the number of women who acquire the STI in question during follow-up; for cumulative incidence, the denominator will be the total number of at-risk women, whereas for incidence density the denominator will be the total number of person years for at-risk women. For each HIV/STI outcome, Poisson regression will be used to determine if group differences are significant. As an indication of effect size, we will calculate the number needed to treat (NNT) for each STI outcome, where NNT represents the number of participants in the intervention who would need to be treated before one fewer person contracted the STI than would be the case had all participants received the comparison condition.

Aim four will be addressed by using FSW outcomes described in aim three, co-varying for fidelity.

We evaluated statistical power to detect main effects and interaction effects in hypothesis three. Our power analyses reveal that we have an excellent chance of detecting significant main effects and interactions greater than 78% of the time. Power is slightly higher for STIs (0.81). The addition of a covariate for fidelity to test hypothesis four does not substantially affect power estimates. Therefore, though our study is not designed to replicate findings from the parent project, we have sufficient power to detect differences between groups.

### Qualitative analyses

Analysts will review both *a priori* concepts that emerge from the quantitative survey data as well as concepts that emerge from field notes, semistructured interviews, and focus groups. The primary issues raised by CBO staff will be identified and coded from each of these data sources. A catalog of domains for such issues will be developed, and the number of individuals raising each issue will be recorded. In each domain, four qualitative interviews that represent variability in influence scores in the domain will be saved for a final coding step. Different interviews may be saved for each domain. During review of the final four interviews in each domain, we will count the number of new issues raised in the interviews that were not raised in previous interviews. If the total number of new issues raised represents more than 20% of the issues raised in prior interviews, further interviews will be conducted to reach conceptual saturation in the domain [44]. We anticipate that few domains will fail to reach conceptual saturation in the first pass. Accuracy of information obtained through the three data collection methods will be assessed through a process of triangulation in which accounts of specific events and behaviors obtained from semi-structured interviews, focus groups, and surveys will be compared [45].

## Discussion

### Limitations

Our protocol represents an unusual hybrid design that is among the first to examine organizational factors in implanting an efficacious intervention, and it is not without limitations. A larger number of study sites would increase power and generalizability. However, this was precluded by funding constraints, something that is likely to be a challenge for many such studies. A related problem is the possible heterogeneity of clinic staff and FSWs between sites, which may affect statistical power. In addition, the use of a waitlist control represents a compromise. As noted, the purpose of this measure was to avoid contamination between experimental conditions, particularly at smaller sites where the number of staff is limited. However, aside from the practical matter of potential loss to follow-up in the experimental condition, it is also possible that the experimental group’s behavior or attitudes regarding safer sex might be influenced during the delay by the mere fact of an intervention with that theme being conducted at the clinic. The realities of resource-limited settings such as Mexico suggested that limiting our study to larger clinics where contamination would be less of a concern (and where the waitlist design could thus be dispensed with) would result in unacceptably great losses to generalizability of our eventual findings. Challenges faced in this project are likely to be faced by other hybrid projects with limited funding in resource-limited settings.

### Innovation and potential impact

This project is innovative in several respects. To our knowledge, it is the first to: determine the extent to which organizational and provider factors influence implementation of an efficacious intervention for FSWs in a resource limited setting, Mexico; potentially provide information useful for implementing efficacious interventions in other developing countries; and create a diverse, interdisciplinary team spanning public health, rehabilitation, addiction treatment, and health economics, which will enhance our understanding of the implementation of an HIV prevention intervention in a developing-world setting.

Given that these CBOs will serve a highly marginalized population (FSWs), they will fill a gap in HIV preventive services, because few funding agencies focus on the needs of FSWs. If our project is successful, our train-the-trainer model could be shown to be a viable and economical approach to implementing and disse-minating efficacious prevention interventions in CBOs. In addition, the US has increasingly recognized the importance of implementation research. For example, NIH has targeted implementation research as a high priority (cf., PAR-07-086: Dissemination and Implementation Research in Health). This project also directly addres-ses several priorities listed in the NIH *Fiscal Year 2010 Trans-NIH Plan for HIV-Related Research*, inclu-ding: supporting international research that examines organizational barriers and facilitators for the adoption and utilization of effective preventive and treatment interventions; supporting domestic and international intervention research; improving capacity of communities to adopt and sustain primary prevention interventions; supporting multidisciplinary programs for intervention research; and conducting collaborative evaluation research to assess the efficacy of strategies to shift HIV care tasks from higher-intensity to lower-intensity trained individuals in resource-limited settings, findings from this project will significantly advance implementation science in this region.

## Competing interests

Gregory A. Aarons is an Associate Editor of *Implementation Science.* The authors have no other competing interests to declare.

## Authors’ contributions

All authors have made substantial contributions to the study conception and design, acquisition of data, and the development and editing of the manuscript, and have given final approval of the version to be published.
